# Supplementary material for: Skin-associated Corynebacterium amycolatum shares cobamides
Source: mSphere. 2024 Dec 18;10(1):e00606-24. doi: 10.1128/msphere.00606-24 (PMC11774034; doi:10.1128/msphere.00606-24)

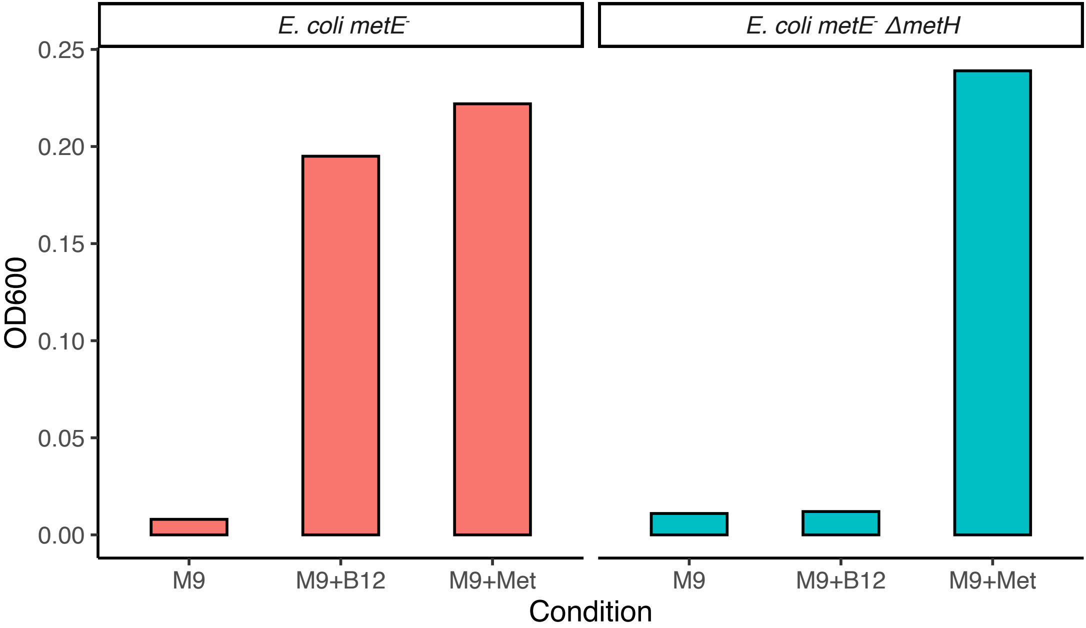

Supplemental Figure 1 E. coli metE- and E. coli metE- ΔmetH (initial OD600=0.01) were cultured for 6 h at 37°C, with or without 0.18 nM cyanocobalamin (B12) or 67.0 μM methionine (Met). Results confirm inability of E. coli metE- ΔmetH to utilize cyanocobalamin for growth.

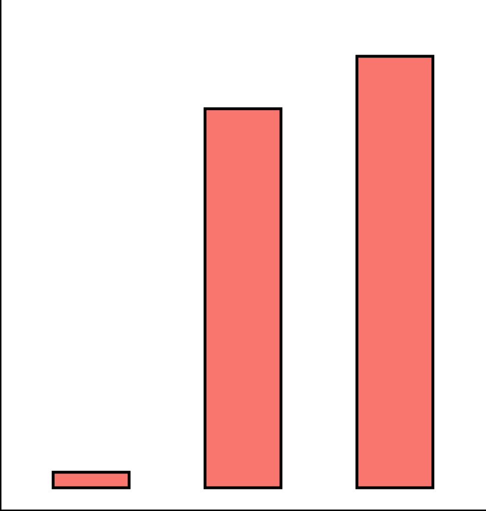

Supplement: Fig. S1 — E. coli metE− ΔmetH is unable to utilize cyanocobalamin for growth. [file msphere.00606-24-s0001.pdf]
