# Supplementary material for: Skin-associated Corynebacterium amycolatum shares cobamides
Source: mSphere. 2024 Dec 18;10(1):e00606-24. doi: 10.1128/msphere.00606-24 (PMC11774034; doi:10.1128/msphere.00606-24)
Supplement: Fig. S2 — Representative cyanocobalamin chromatogram of cell extract from C. amycolatum LK19 WT. [file msphere.00606-24-s0002.pdf]

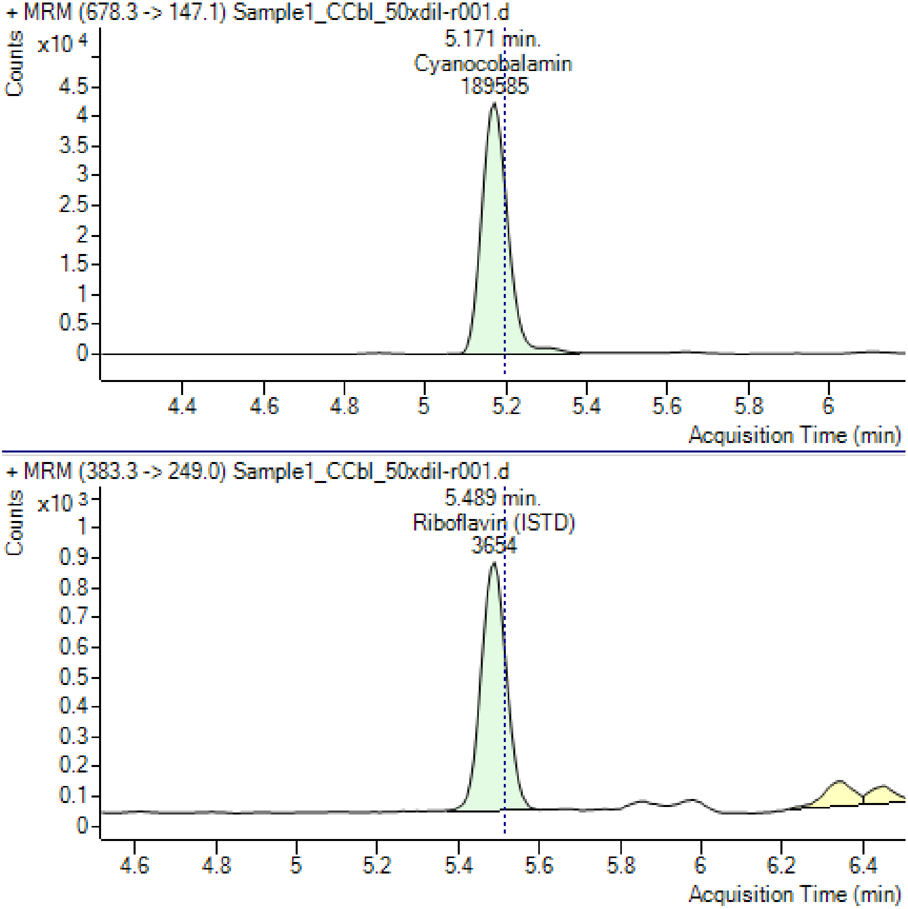


Supplemental Figure 2. Representative cyanocobalamin chromatogram of cell extract from C. amycolatum LK19 WT (250 nM CoCl2). Sample was diluted 50-fold on column to fall within the standard curve. Top panel shows the cyanocobalamin levels in the sample, and bottom panel showss the internal standard. Cyanocobalamin was quantified in this sample at 14.931 µg/mL (11.02 µM). The dotted line represents the mid-point of the chromatogram.
