# Supplementary material for: Skin-associated Corynebacterium amycolatum shares cobamides
Source: mSphere. 2024 Dec 18;10(1):e00606-24. doi: 10.1128/msphere.00606-24 (PMC11774034; doi:10.1128/msphere.00606-24)
Supplement: Fig. S3 — E. coli metE−, E. coli metE− ΔmetH, and C. amycolatum growth in the liquid co-culture system. [file msphere.00606-24-s0003.pdf]

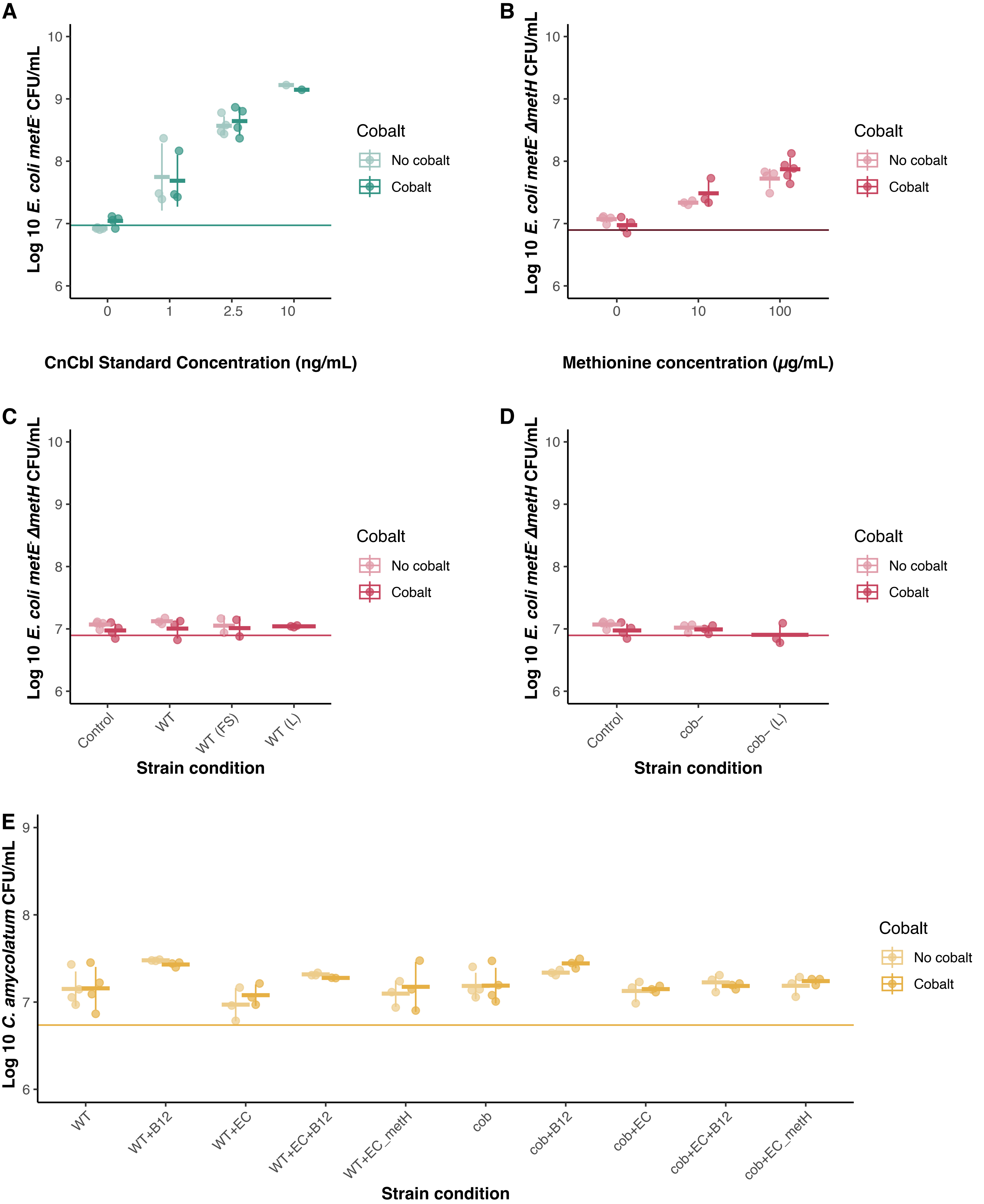

Supplemental Figure 3. (A) E. coli metE- (blue) was cultured with 0, 1, and 2.5, and 10 ng/mL (0, 0.74, 1.84, 7.38 nM) cyanocobalamin standards (CNCbl), and (B) E. coli metE- ΔmetH (pink) was cultured with 0, 10, and 100 μg/mL (0, 67.0, 670.2 μM) methionine to validate expected growth response of each strain, quantified in CFU/mL. Growth of E. coli metE- is proportional to the concentration of cobamides or methionine in the medium, and growth of E. coli metE- ΔmetH is proportional to the concentration of only methionine, thus distinguishing between cobamides and methionine in supporting E. coli growth. C. amycolatum WT (WT) (C) and C. amycolatum cob- (cob-) (D) were co-cultured in minimal medium with E. coli metE- Δ metH under low (0 nM) or high (250 nM) cobalt concentrations. (E) C. amycolatum CFU/mL was measured to demonstrate growth across each strain condition. n= at least 3 biological replicates, averaged across 3 technical replicates, for each condition (3 technical replicates across 3 biological replicates). Solid lines represent the measured inoculum for E. coli metE-, E. coli metE- ΔmetH, or C. amycolatum averaged across all replicates. CNCbl = 2.5 ng/mL (1.84 nM) cyanocobalamin, FS = filter-sterilized C. amycolatum cell suspension, L = lysed C. amycolatum cell suspension.
