# Supplementary material for: Skin-associated Corynebacterium amycolatum shares cobamides
Source: mSphere. 2024 Dec 18;10(1):e00606-24. doi: 10.1128/msphere.00606-24 (PMC11774034; doi:10.1128/msphere.00606-24)
Supplement: Fig. S4 — E. coli metE− and E. coli metE− ΔmetH growth in the solid medium co-culture system. [file msphere.00606-24-s0004.pdf]

A

Condition	*E. coli* metE-	*E. coli* metE- ΔmetH
C. amycolatum WT **(B)**	Fig. 4	-
C. amycolatum cob⁻ **(C)**	Fig. 4	-
Control **(D,E)**	-	-
100 ng/mL CnCbl **(F)**	+++	-
10 ng/mL CnCbl **(F)**	+	-
1 mg/mL methionine **(G)**	+++	+++
100 µg/mL methionine **(G)**	+	+

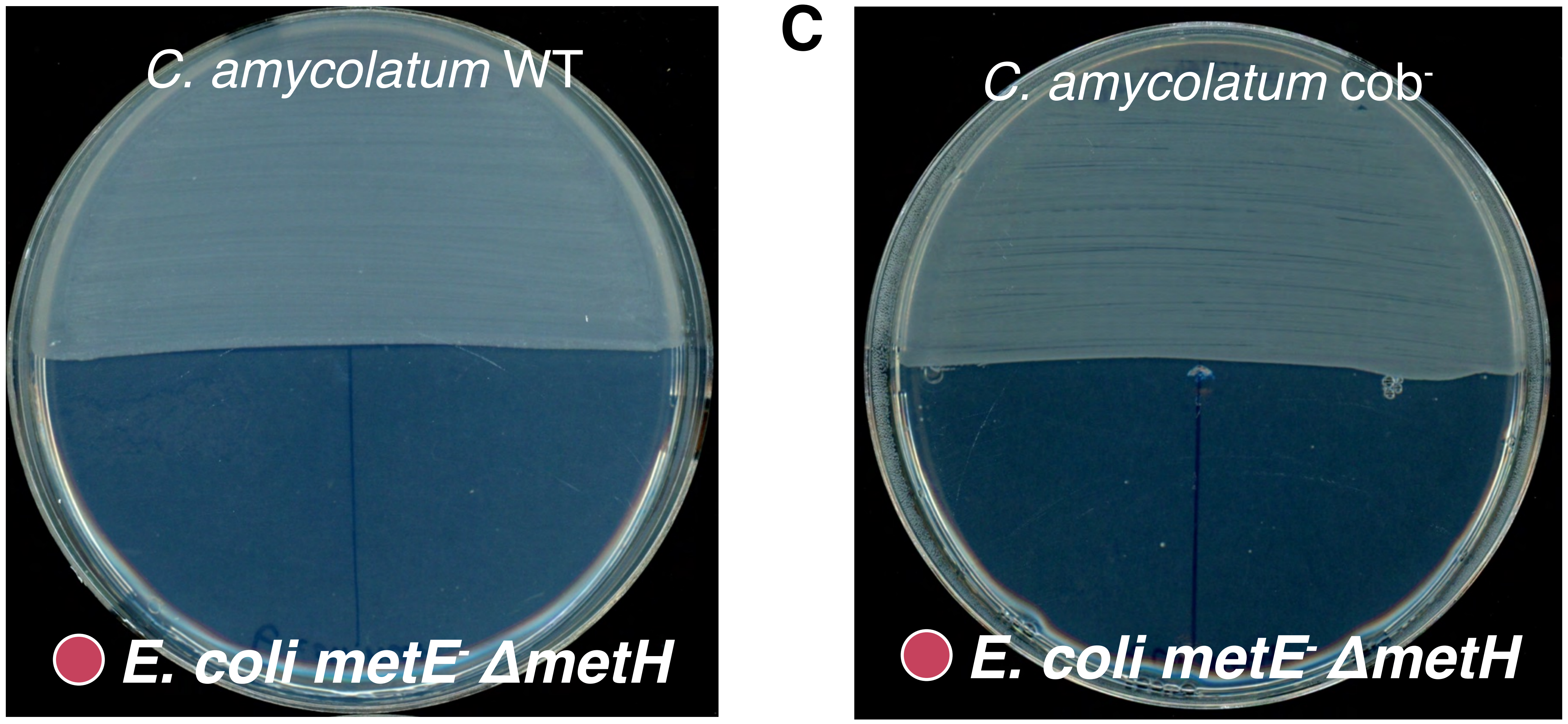

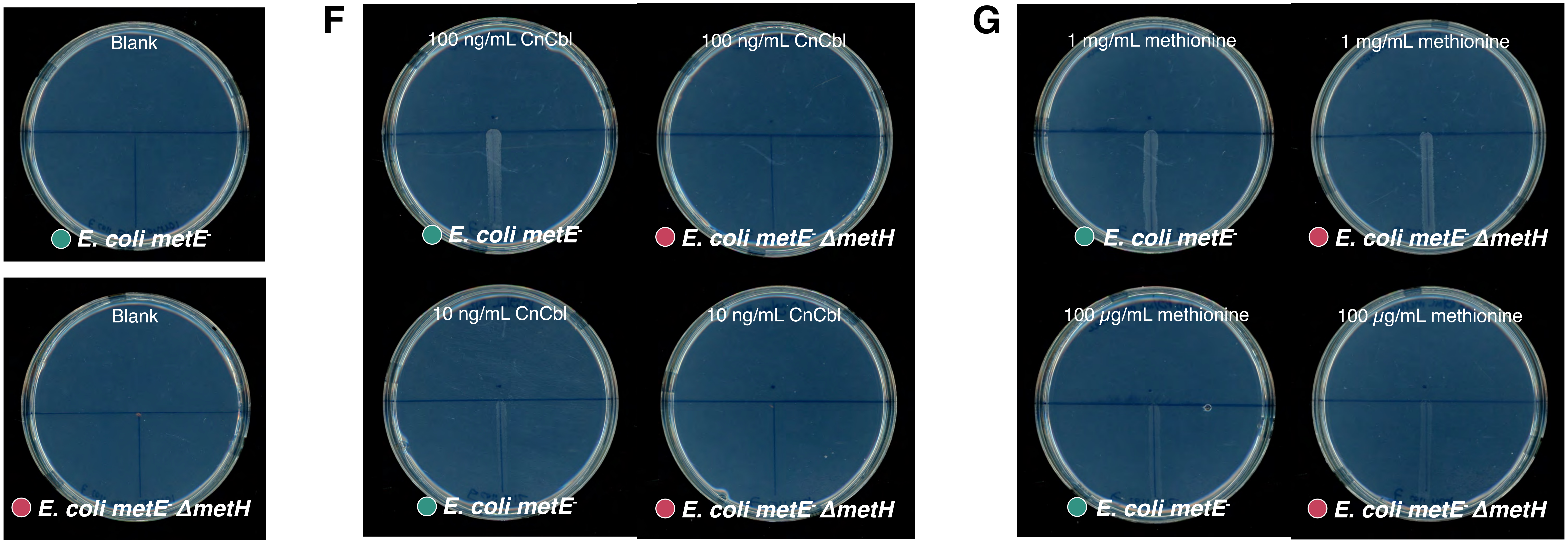

Supplemental Figure 4. C. amycolatum WT cell suspension, C. amycolatum cob- cell suspension, or controls (CNnCbl or methionine) were added to half of a minimal medium plate and incubated for 3 days, after which E. coli metE- (blue) or E. coli metE- ΔmetH (pink) was streaked out on the adjacent side of the plate. E. coli growth was recorded after 24 h. Growth summary for all conditions is indicated in (A). - indicates no growth and +, ++, +++ indicate increasing levels of growth, respectively. (B) C. amycolatum WT lawn and (C) C. amycolatum cob- lawn with E. coli metE- ΔmetH. Blank controls for (D) E. coli metE- and (E) E. coli metE- ΔmetH. (F) E. coli metE- and (G) E. coli metE- ΔmetH growth response to CNnCbl and methionine standards, respectively.
