# Supplementary material for: Skin-associated Corynebacterium amycolatum shares cobamides
Source: mSphere. 2024 Dec 18;10(1):e00606-24. doi: 10.1128/msphere.00606-24 (PMC11774034; doi:10.1128/msphere.00606-24)
Supplement: Fig. S5 — E. coli metE− growth in response to C. amycolatum co-culture under different cobalt conditions. [file msphere.00606-24-s0005.pdf]

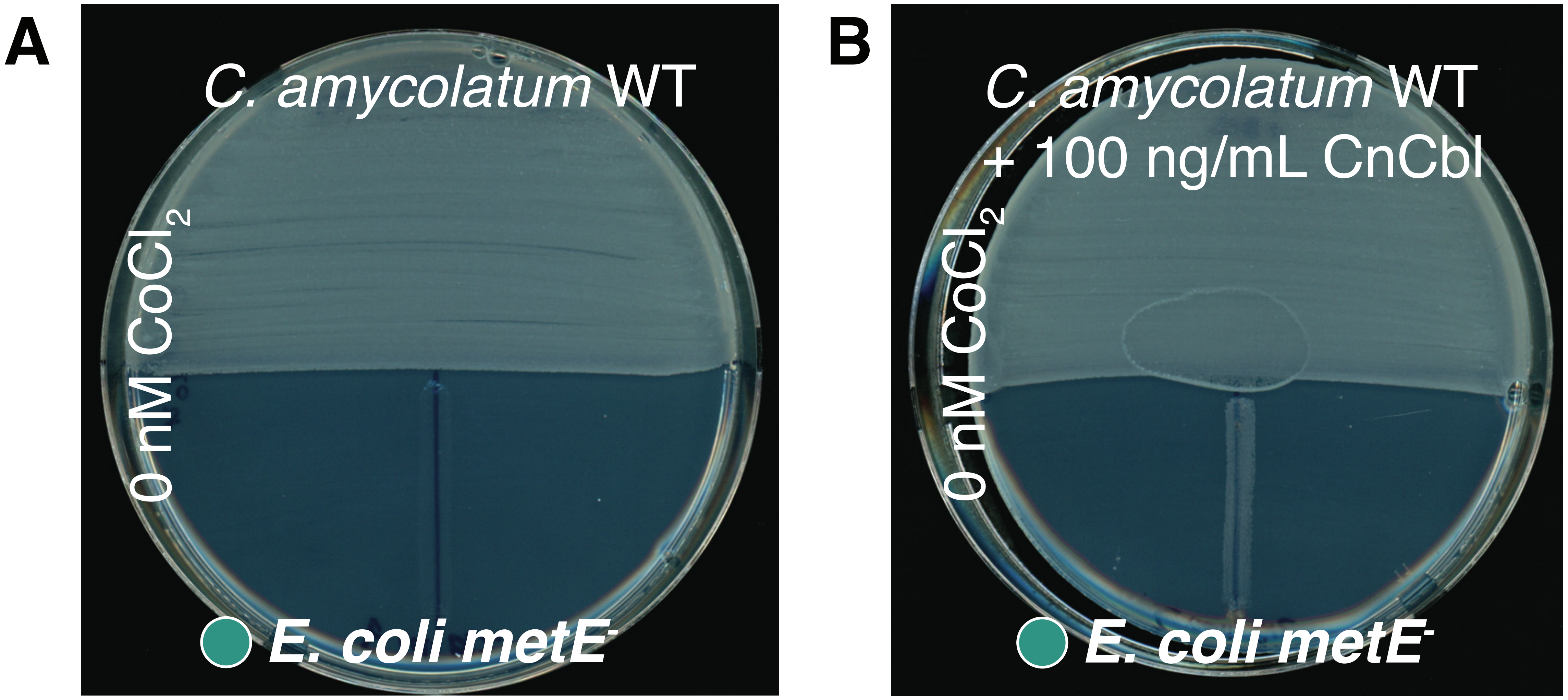

F

Condition	0 nM $CoCl_2$	250 nM $CoCl_2$
C. amycolatum WT	-	++
C. amycolatum WT + 100 ng/mL CnCbl	+++	ND
Control	-	-
10 ng/mL CnCbl	+	+
100 ng/mL CnCbl	+++	+++

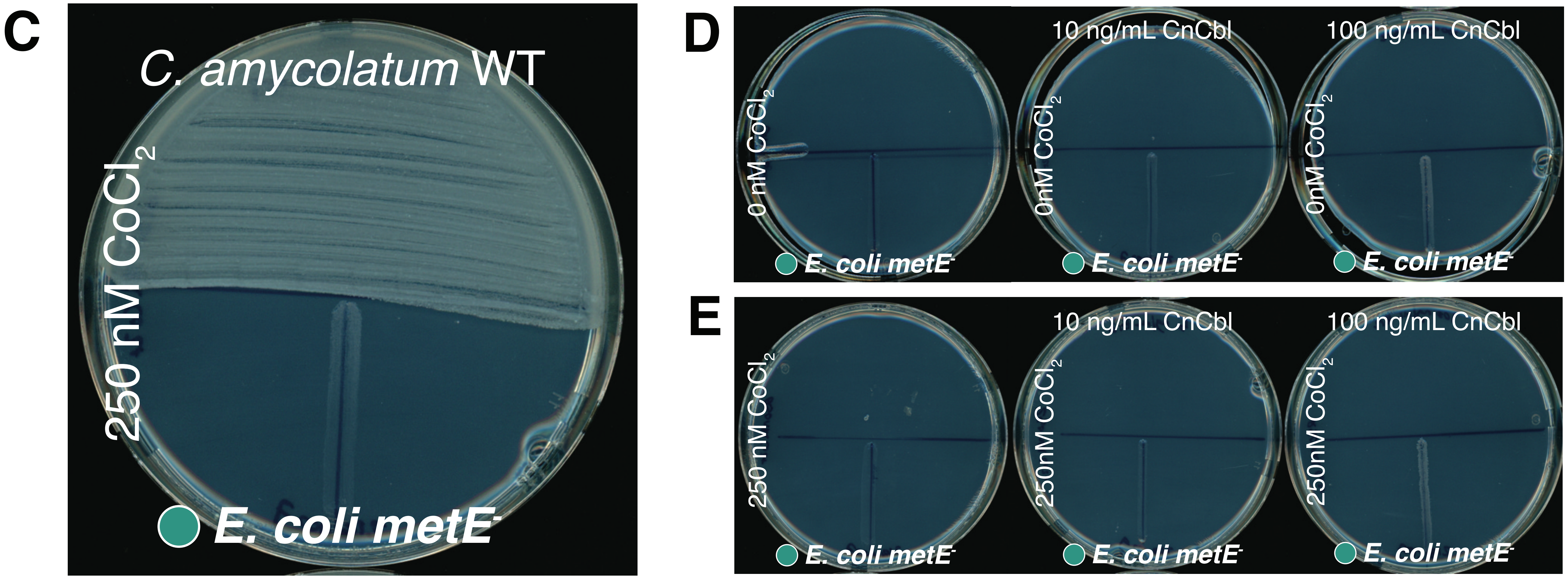

Supplemental Figure 5. C. amycolatum WT cell suspension was added to half of a minimal medium plate (0 nM or 250 nM CoCl2) and incubated for 3 days, after which E. coli metE- (blue) was streaked out on the adjacent side of the plate. E. coli growth was recorded after 24 h. Growth summary for all conditions are indicated in F). - indicates no growth and +, ++, +++ indicate increasing levels of growth, respectively. (A) C. amycolatum WT lawn on medium with 0 nM CoCl2 and (B) C. amycolatum WT lawn with 0 nM CoCl2 and 73.8 nM CNCbl spotted on top. (C) C. amycolatum WT lawn on medium with 250 nM CoCl2. CNCbl standards (0, 7.38, and 73.8 nM) + E. coli metE- on minimal medium plates with (D) 0 nM CoCl2 or (E) 250 nM CoCl2.
