# Supplementary material for: Skin-associated Corynebacterium amycolatum shares cobamides
Source: mSphere. 2024 Dec 18;10(1):e00606-24. doi: 10.1128/msphere.00606-24 (PMC11774034; doi:10.1128/msphere.00606-24)
Supplement: Fig. S6 — Generation of cobamide-deficient C. amycolatum. [file msphere.00606-24-s0006.pdf]

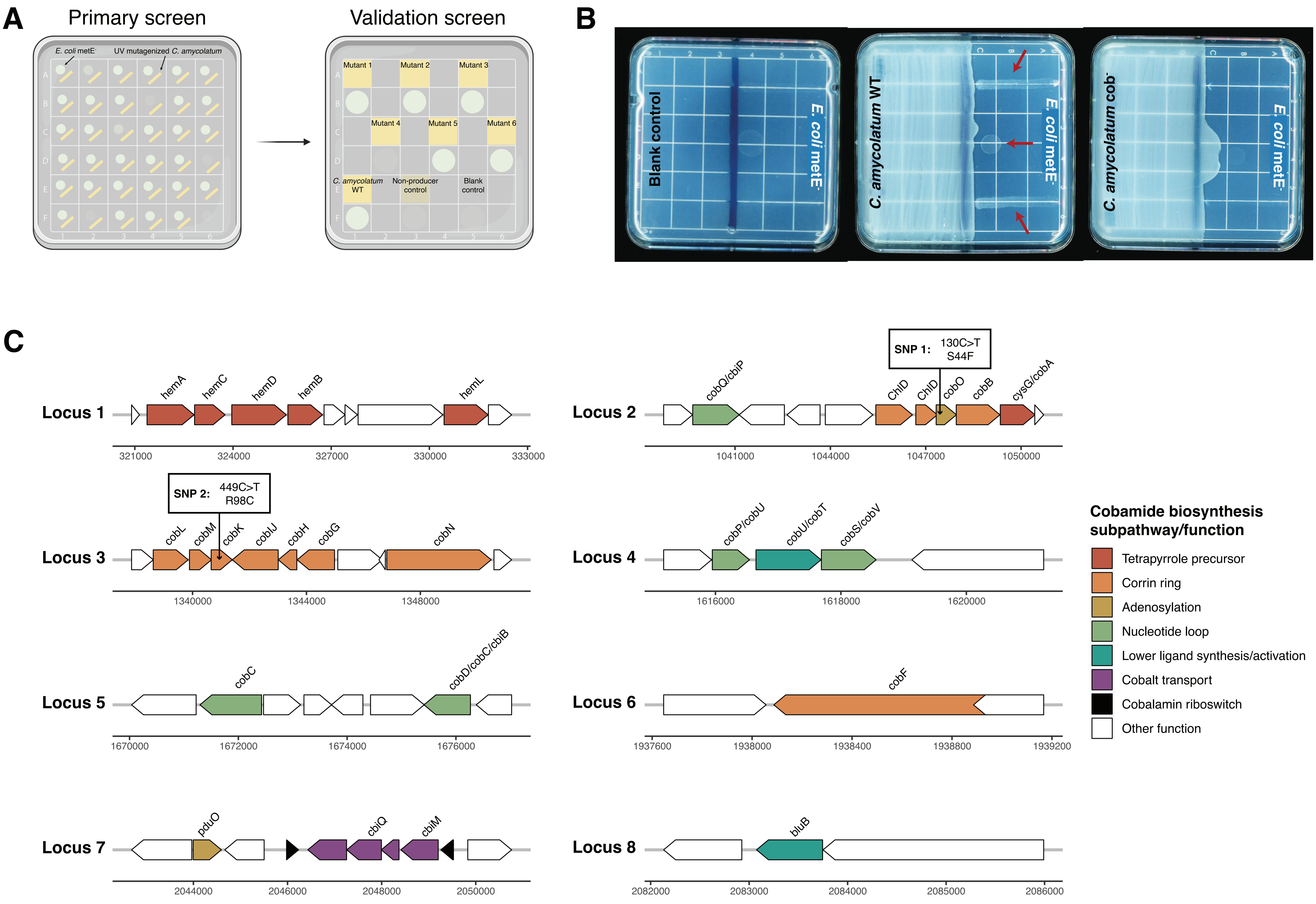

Supplemental Figure 6 (A) A novel screen was designed to test for biosynthesis of cobamides by C. amycolatum using the indicator strain E. coli metE-. UV mutagenized colonies of C. amycolatum were patched onto minimal medium, followed by spotting of E. coli metE- adjacent to the patches, after which growth of E. coli was assessed after 18 hours. Potential mutants were then validated using a similar assay, with growth of E. coli in response to the potential mutants compared to the following controls: C. amycolatum WT, a non-cobamide producer C. jeikeium, and a blank control. (B) E. coli metE- was grown on minimal medium plates alone, with C. amycolatum WT, and with C. amycolatum cob- and assessed for growth to indicate presence of cobamides. (C) Whole-genome sequencing of C. amycolatum cob- was performed to identify location of mutations affecting cobamide biosynthesis. Loci of cobamide biosynthesis genes are shown, with genes colored by cobamide biosynthesis subpathway or function. Identified SNPs are highlighted in loci 2 and 3.
