# Supplementary material for: Skin-associated Corynebacterium amycolatum shares cobamides
Source: mSphere. 2024 Dec 18;10(1):e00606-24. doi: 10.1128/msphere.00606-24 (PMC11774034; doi:10.1128/msphere.00606-24)
Supplement: Fig. S7 — cobK and cobO sequences and structures. [file msphere.00606-24-s0007.pdf]

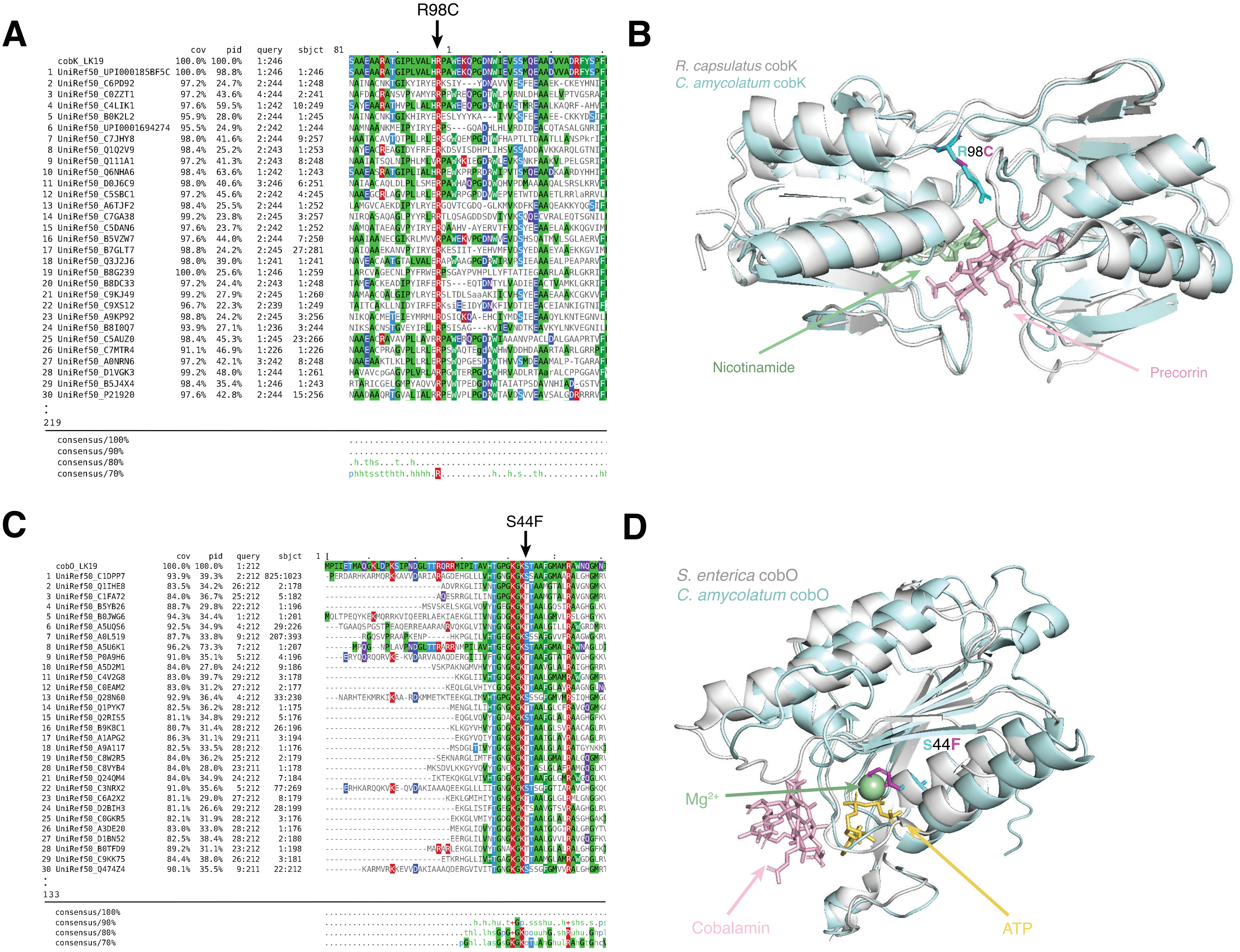


Supplemental Figure 7. Sequence homologs of (A) cobK and (C) cobO were detected by querying C. amycolatum WT protein sequences against the Uniref50 sequence database using PSI-BLAST through the Phyre2 pipeline. Sequences are visualized with MView, with residues colored by identity. The top 30 alignments are shown. The consensus sequence is based on all alignments (up to 1000). Alphafold2 was used to predict the structure of (B) cobK and (D) cobO. Structures were then aligned to the predicted ternary structures of cobK from Rhodobacter capsulatus (PDB code: 4X7G) and cobO from Salmonella enterica (PDB code: 1G64). C. amycolatum WT structures are shown in light blue and PDB structures are shown in white. Missense3D was then used to predict the structure of C. amycolatum cob- cobK and cobO. Residue changes are shown in (B) for cobK and (D) for cobO, with the wild-type residue depicted in cyan and the cob- residue in magenta.
