# Supplementary material for: Skin-associated Corynebacterium amycolatum shares cobamides
Source: mSphere. 2024 Dec 18;10(1):e00606-24. doi: 10.1128/msphere.00606-24 (PMC11774034; doi:10.1128/msphere.00606-24)
Supplement: Fig. S9 — C. amycolatum WT and cob− morphology and growth characteristics. [file msphere.00606-24-s0009.pdf]

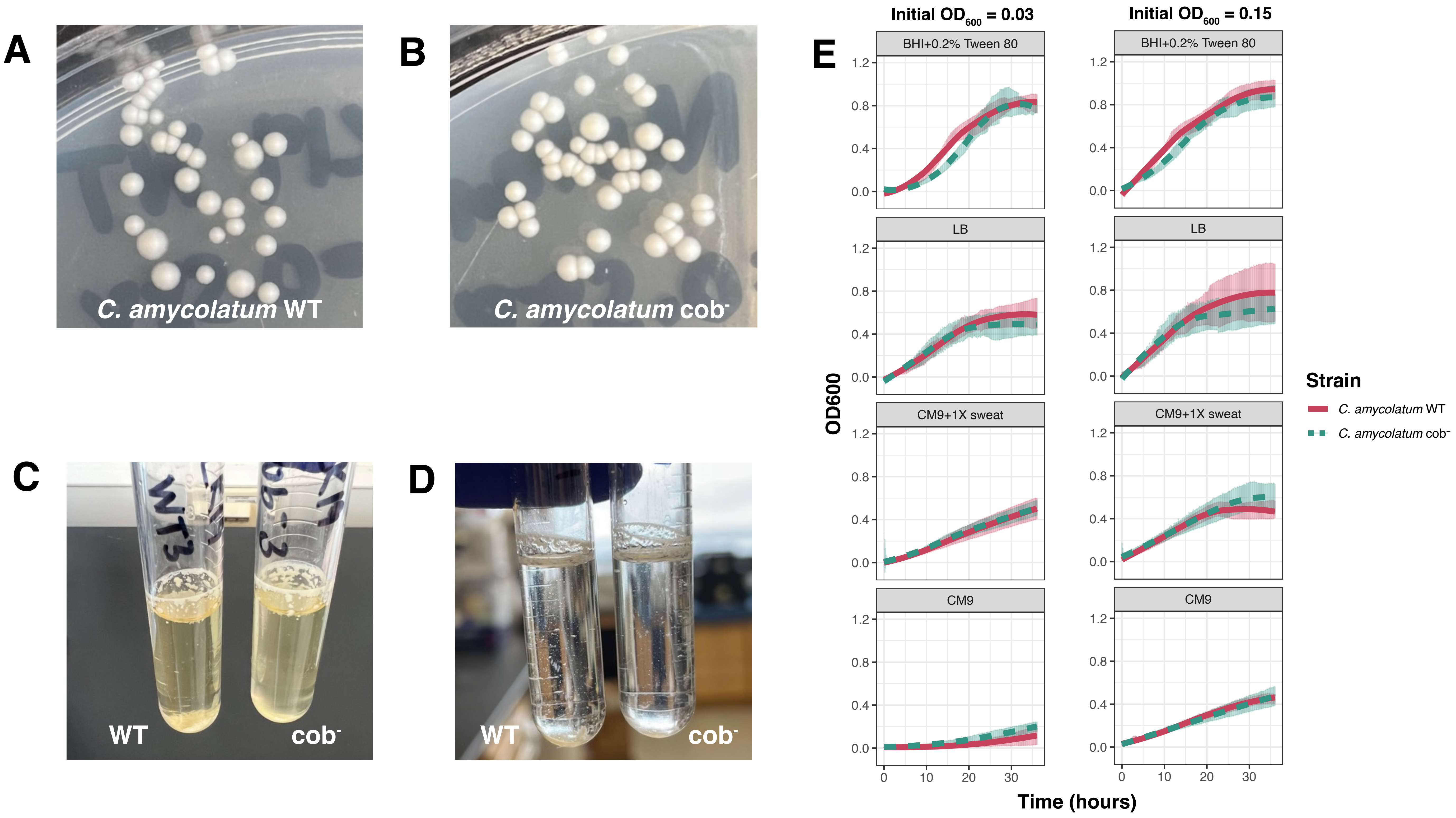

Supplemental Figure 9 (A) C. amycolatum WT and (B) C. amycolatum cob- colony morphology on TSA + 0.2% Tween 80 plates. C. amycolatum WT and C. amycolatum cob- morphology when grown in (C) liquid rich medium (BHI + 0.2% Tween 80) or (D) minimal medium (CM9 + artificial sweat). (E) Cells from overnight cultures of C. amycolatum WT and C. amycolatum cob- were washed and inoculated to an initial OD600 of 0.03 or 0.15 in BHI + 0.2% Tween 80, LB, CM9 + 1X sweat, or CM9. Growth was measured over 36 hours for each strain by collecting OD600 measurements.
