# Supplementary material for: Skin-associated Corynebacterium amycolatum shares cobamides
Source: mSphere. 2024 Dec 18;10(1):e00606-24. doi: 10.1128/msphere.00606-24 (PMC11774034; doi:10.1128/msphere.00606-24)
Supplement: Fig. S8 — Sequence homologs of miaB, murB, pepN, DUF3329, and 4Fe4S dicluster domain protein. [file msphere.00606-24-s0008.pdf]

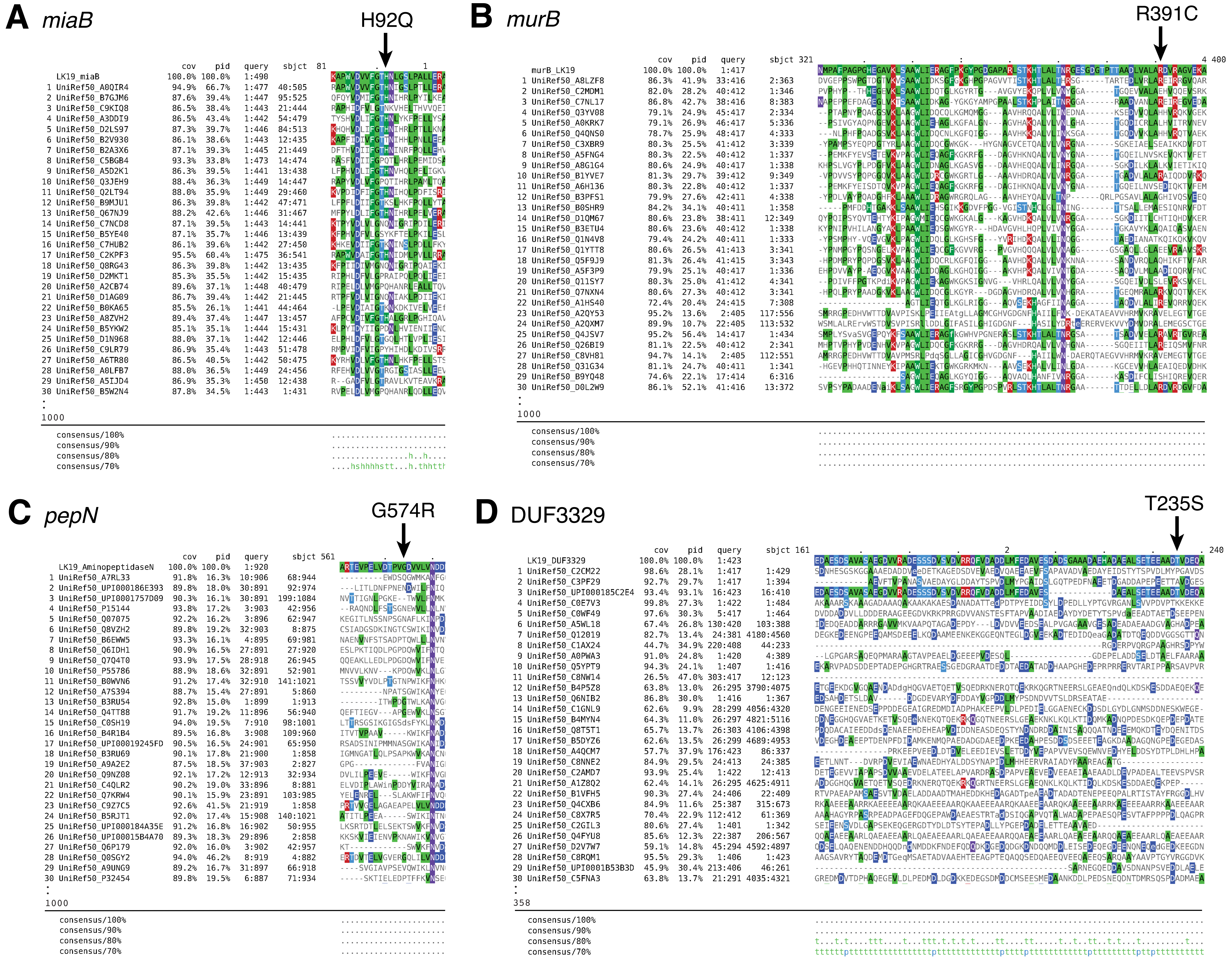

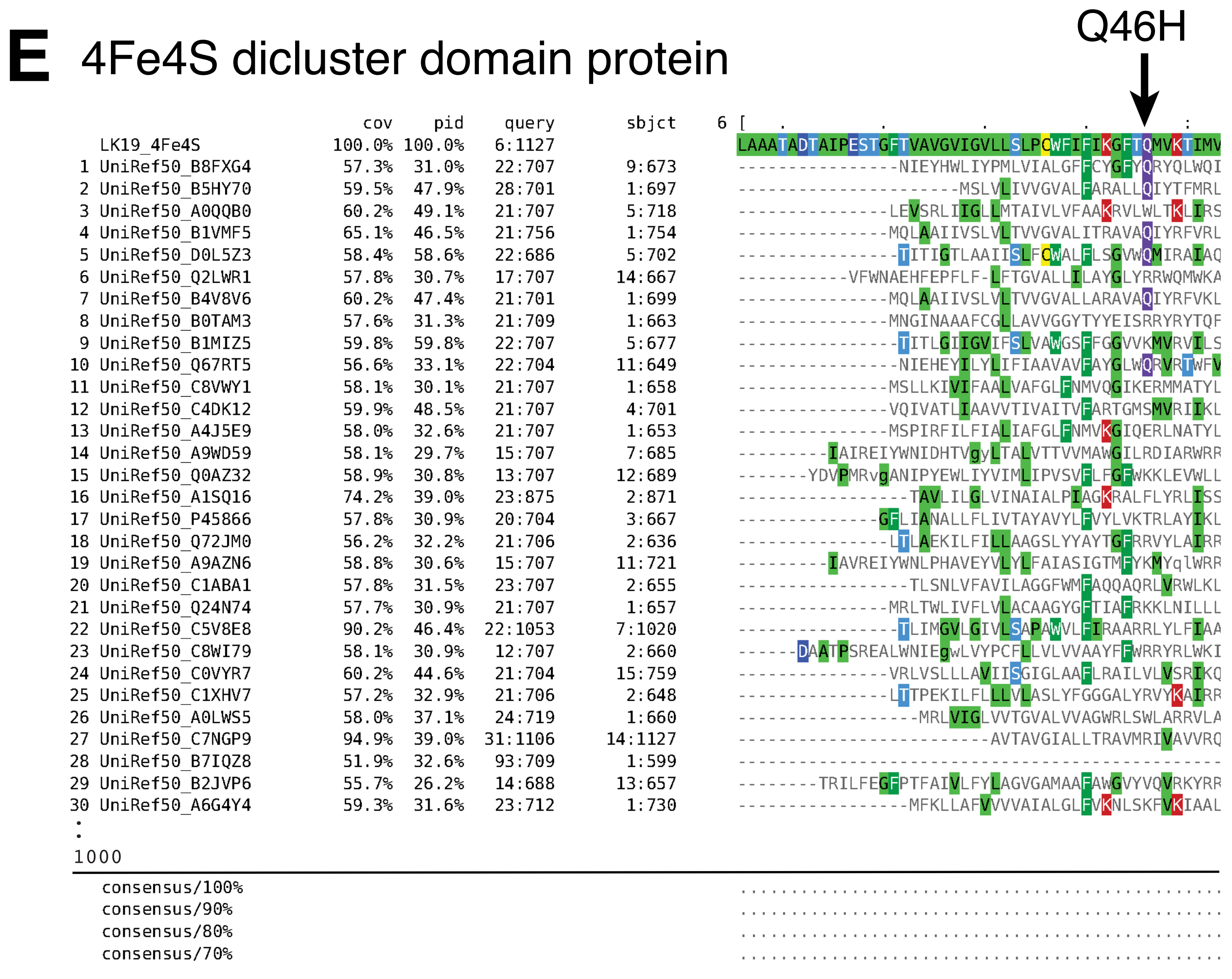

Supplemental Figure 8. Sequence homologs of (A) miaB, (B) murB, (C) pepN, (D) DUF3329, and (E) 4Fe4S dicluster domain protein were detected by querying C. amycolatum WT protein sequences against the Uniref50 sequence database using PSI-BLAST through the Phyre2 pipeline. Sequences are visualized with MView, with residues colored by identity. The top 30 alignments are shown. The consensus sequence is based on all alignments (up to 1000).
